# New first trimester circulating angiogenic biomarkers in predicting early-onset and late-onset fetal growth restriction: a case-control study

**DOI:** 10.1186/s12884-025-07558-4

**Published:** 2025-05-10

**Authors:** Xiaoyi Bai, Wei Li, Wenjing Ding, Oi Ka Chan, Maran Bo Wah Leung, So Ling Lau, Daljit Singh Sahota, Chi Chiu Wang, Tak Yeung Leung

**Affiliations:** 1 Department of Obstetrics and Gynaecology, Faculty of Medicine, The Chinese University of Hong Kong, Shatin, Hong Kong; 2 Department of Gynaecology, Guangdong Provincial People’s Hospital (Guangdong Academy of Medical Sciences), Southern Medical University, Guangzhou, China; 3 Department of Laboratory Medicine, Maternity and Child Healthcare Hospital of Nanshan District, Shenzhen, China; 4 Reproduction and Development, Li Ka Shing Institute of Health Sciences, The Chinese University of Hong Kong, Shatin, Hong Kong; 5 School of Biomedical Sciences, The Chinese University of Hong Kong, Shatin, Hong Kong; 6 The Chinese University of Hong Kong-Baylor College of Medicine Joint Centre for Medical Genetics, The Chinese University of Hong Kong, Shatin, Hong Kong

**Keywords:** Angiogenic biomarker, Fetal growth restriction, Platelet-derived growth factor, Placenta growth factor, Pregnancy-associated plasma protein A, Soluble fms-like tyrosine kinase-1, Soluble neuropilin-1, Soluble platelet and endothelial cell adhesion molecule 1

## Abstract

**Background:**

First trimester prediction of fetal growth restriction (FGR) remain suboptimal. We aimed to search for new circulating angiogenic biomarkers for improvement.

**Methods:**

This case-control study compared 73 singleton pregnancies with early or late-onset FGR based on Delphi consensus and 73 matched normal controls. Their maternal serum samples stored during 11–13 weeks were retrieved for measurement of 36 angiogenic biomarkers by MILLIPLEX^®^ human angiogenesis magnetic bead panels. Those biomarkers that showed significant differences between the study groups were further analysed with receiver operating characteristic (ROC) curve.

**Results:**

In the early-onset FGR group, log_10_MoM of soluble neuropilin-1 (sNRP-1: 0.08 ± 0.11 vs. 0.00 ± 0.09, *P* < 0.001) and log_10_MoM of soluble platelet and endothelial cell adhesion molecule 1 (sPECAM-1: 0.05 ± 0.06 vs. 0.00 ± 0.09, *P* < 0.05) were significantly higher than the control group, while log_10_MoM of platelet-derived growth factor AB/BB (PDGF-AB/BB: -0.08 ± 0.13 vs. 0.00 ± 0.16, *P* < 0.05) and PAPP-A (-0.15 ± 0.28 vs. 0.05 ± 0.23, *P* < 0.001) were lower. Their combination achieved the highest area under the ROC curve (AUC) of 0.83 (95% CI: 0.74–0.95) with a higher sensitivity than that of PAPP-A alone (61.5% vs. 30.8% at 10% false positive rate, *P* < 0.001). Concerning the late-onset FGR group, only log_10_MoMs of sFlt-1 (-0.12 vs. 0.00, *P* < 0.001) and PAPP-A (-0.07 vs. 0.05, *P* < 0.05) were lower than the control group, but their AUC was only 0.68 (95% CI:0.59–0.78).

**Conclusions:**

Three new first trimester biomarkers, sNRP-1, sPECAM-1 and PDGF-AB/BB are predictive of subsequent development of early-onset FGR.

**Supplementary Information:**

The online version contains supplementary material available at 10.1186/s12884-025-07558-4.

## Background

Fetal growth restriction (FGR) affects 5–10% of pregnancies and is one of the leading causes of preterm birth, stillbirth and neonatal death, as well as various neonatal short term and long term morbidities [1–4]. The causes of FGR are diverse and include maternal, fetal and placental disorders, such as fetal chromosomal or genetic abnormalities, maternal hypertensive pregnancy diseases, and uteroplacental vascular insufficiency. While high risk pregnancies with a known cause or risk factors are often closely monitored sonographically, and therefore are diagnosed and managed at an earlier stage, low risk pregnancies are often screened by symphysis-fundal height measurement only. Although symphysis-fundal height measurement is a simple method to screen FGR, it is notorious for its low sensitivity and high false positive rate [5]. Serial sonographic measurement of fetal biometrics allows more accurate assessment of fetal growth, but it is labor intensive and hence is not cost-effective as a routine for low risk population. Routine single fetal sonographic measurement at 35–37 gestational weeks was proposed but its value in detecting FGR remains controversial [6–9].

First trimester maternal serum levels of biochemical markers such as pregnancy-associated plasma protein-A (PAPP-A) and placental growth factor (PlGF), soluble fms-like tyrosine kinase 1 (sFlt-1) are known to be lower in those pregnancies which subsequently developed to FGR. Hence it may be used to triage pregnancies at risk of FGR. It can be done at the same time as screening for Down syndrome or pre-eclampsia during the first trimester. Yet the predictive performance of these markers are still far from satisfactory for clinical use [10–13].

Hence there is a need to discover new biomarkers. As imbalanced angiogenesis is observed in the pathophysiology of FGR [14], the aim of this study was to search for new first trimester angiogenic biochemical markers in maternal circulation which may be associated with subsequent development of FGR, and to investigate their potential in predicting FGR.

## Methods

### Setting

This was a case-control study in a tertiary hospital from March 2019 to January 2022. This study was approved by the ethics committee of the Joint Chinese University of Hong Kong - New Territories East Cluster Clinical Research Ethics Committee (CREC reference No.2020.313). In the study unit, every pregnant woman had been offered routine first trimester combined screening for trisomies since 2010 [15]. During 11^+ 0^ weeks to 13^+ 6^ weeks, ultrasound was performed to confirm the gestational age determined by crown rump length [16] and to measure nuchal translucency. Maternal peripheral blood was sampled for PAPP-A and free beta human chorionic gonadotropin assays. A portion of the maternal serum samples was stored at -80℃ for research purposes with maternal consent.

Clinical data were retrievable from the study center’s medical system, including maternal demographical data such as maternal age, maternal height and weight, parity, obstetric history, method of conception, smoking status, pre-existing medical history; pregnancy outcomes such as gestation at delivery, delivery mode, neonatal sex and birthweight, Apgar score, umbilical arterial blood gas results and neonatal intensive care unit admission. Birthweight (BW) Z score were calculated = (observed BW– mean BW of the completed gestational weeks) / standard deviation (SD) from the local reference population [17].

### Subjects

#### FGR cohort

Pregnant women then underwent routine antenatal check-ups with fundal height measurement, every 4 weeks since 16 gestational weeks, then every bi-weekly since 28 weeks, and weekly since 37 weeks. Those women with fundal height were smaller than expected were followed by fetal sonogram. We followed the Delphi consensus in defining early-onset and late-onset FGR as shown in Table 1 [18]. The centile of abdominal circumference and estimated fetal weight were derived from our local population-based reference [19]. We excluded multiple pregnancies, and pregnancies with fetal genetic or structure abnormalities, intrauterine death, miscarriage, antiphospholipid syndrome, diabetes mellitus, and hypertensive disorders in pregnancy. Pregnancies that were screened positive for preterm pre-eclampsia by uterine artery Doppler and PlGF (calculated risk greater than 1:100) [20] or treated with aspirin were also excluded. Confirmed FGR cases were further monitored for fetal growth and well-being, which included fetal biometric and amniotic fluid index measurement, Doppler measurement of flows in umbilical artery, middle cerebral artery and ductus venosus, and cardiotocogram. Fetuses that were at high risk of intrauterine death, such as those with absent or reversed ductus venosus a-wave, absent or reversed umbilical artery end-diastolic velocity, abnormal short-term variation cardiotocogram, were counselled for early delivery. In case of preterm birth, antenatal treatment with glucocorticoids (before 34 gestational weeks) or magnesium sulphate (before 32 weeks) was considered respectively for fetal lung maturation and prophylaxis against cerebral palsy. Induction of labor and the mode of delivery depended on the severity of FGR, individual maternal, fetal and obstetric factors according to the unit’s clinical management protocol. The diagnosis of FGR were further confirmed after birth with the sex specific BW of < 10th centile of local reference population [17].

**Table 1:** The definitions of early-onset and late-onset fetal growth restriction (FGR) based on Delphi consensus [18]

Early-onset FGR	Late-onset FGR
GA < 32 weeks, without congenital anomalies	GA ≥ 32 weeks, without congenital anomalies
AC/EFW < 3rd centile or UA-AEDF	AC/EFW < 3rd centile
Or	Or at least two out of three of following
1.AC/EFW < 10th centile combined with	1. AC/EFW < 10th centile
2.UtA-PI > 95th centile and/or	2.AC/EFW crossing centile > 2 quartiles
3.UA-PI > 95th centile	3.CPR < 5th centile or UA-PI > 95th centile

*AC*, fetal abdominal circumference; *AEDF*, absent end-diastolic flow; *CPR*, cerebroplacental ratio; *EFW*, estimated fetal weight; *GA*, gestational age; *PI*, pulsatility index; *UA*, umbilical artery; *UtA*, uterine artery

#### Normal controls

Each of the included FGR cases was matched with a control case which also underwent first trimester combined screening. The match was based on the maternal ethnicity, age (± 3 years), parity (nulliparous or multiparous), maternal weight (± 5 kg), calendar date at first trimester Down syndrome screening (± 30 days) and gestation age at blood sampling (± 7 days). Their babies’ birthweights were between 25th and 75th centile corrected for neonatal sex using the same population reference [17].

### Measurement of angiogenic biomarkers

The stored maternal serum samples of both FGR subjects and their matched controls were retrieved and thawed completely at 4℃ for the measurement of the levels of angiogenic biomarkers. None of them was previously thawed and refrozen. We measured the angiogenic biomarkers using MILLIPLEX^®^ human Angiogenesis Magnetic Bead Panel 1 (HAGP1MAG-12 K, Millipore Corporation, MA, USA) and Panel-2 (HANG2MAG-12 K, Millipore Corporation, MA, USA), which has been applied in the oncological studies [21]. The two panels respectively included 17 and 19 angiogenic biomarkers, such as PlGF and sFlt-1, which are known markers for pre-eclampsia (Table 2). These panels are based on the Luminex^®^ xMAP^®^ technology, performing immunoassays on the surface of fluorescent-coded microsphere beads. Similar to sandwich-based ELISA, beads are coated with specific antibodies and could detect multiple analytes simultaneously. The limit of detection, inter-assay and intra-assay coefficients of variation for each biomarker from these panels are listed in Supporting Information Table S1.

**Table 2:** The biomarkers included in angiogenesis magnetic bead Panel-1 (HAGP1MAG-12 K) and Panel-2 (HANG2MAG-12 K)

Angiogenesis Magnetic Bead Panel-1		Angiogenesis Magnetic Bead Panel-2	
Biomarkers	Abbreviations	Biomarkers	Abbreviations
Angiopoietin 2	ANG-2	Angiostatin	ANGST
Bone Morphogenetic Protein 9	BMP-9	Osteopontin	OPN
Epidermal Growth Factor	EGF	Platelet-derived Growth Factor AB/BB	PDGF-AB/BB
Endoglin	ENG	soluble AXL Receptor Tyrosine Kinase	sAXL
Endothelin 1	ET-1	soluble Stem Cell Factor Receptor c-kit	sc-Kit
Fibroblast Growth Factor 1	FGF-1	soluble Hepatocyte Growth Factor Receptor	sc-Met
Fibroblast Growth Factor 2	FGF-2	soluble Epidermal Growth Factor receptor	sEGFR
Follistatin	FS	soluble fms-like Tyrosine Kinase-1	sFlt-1/sVEGFR-1
Granulocyte Colony-stimulating Factor	G-CSF	soluble fms-like Tyrosine Kinase-4	sFlt-4/sVEGFR-3
Heparin Binding EGF-like Growth factor	HB-EGF	soluble Human Epidermal Growth Factor Receptor 2	sHER2
Hepatocyte Growth Factor	HGF	soluble Human Epidermal Growth Factor Receptor 3	sHER3
Interleukin 8	IL-8	soluble interleukin 6 Receptor alpha	sIL-6Ra
Leptin	LEP	soluble Neuropilin-1	sNRP-1
Placenta Growth Factor	PlGF	soluble Platelet and Endothelial Cell Adhesion Molecule 1	sPECAM-1
Vascular Endothelial Growth Factor A	VEGF-A	soluble E-selectin	sSELE
Vascular Endothelial Growth Factor C	VEGF-C	soluble Tie 2	sTie-2
Vascular Endothelial Growth Factor D	VEGF-D	soluble urokinase -type Plasminogen Activator Receptor	su-PAR
		soluble Vascular Endothelial Growth Factor Receptor 2	sVEGFR-2
		Thrombospondin-2	TSP-2

All procedures were carried out according to the manufacturer’s instructions. The absolute concentrations of angiogenic biomarkers were measured by panels run on Luminex^®^ Bio-Plex™ 200 system (Luminex company, Austin, TX, USA) and the concentration of biomarkers were calculated using the five-parameter logistic method by the Bio-Plex Manager software. Results were expressed in picogram per milliliter (pg/ml).

Log_10_ multiple of expected normal median (MoM) level of each angiogenic biomarker was calculated through following steps. Firstly, the crude measurements of each biomarker were log_10_ transformed to make its distribution Gaussian. Multivariate regression analysis were performed to determine whether log_10_ transformed levels of each individual angiogenic biomarker was dependent on gestational age (weeks), maternal age (years), height (cm), weight (kg), smoking status at conception (yes or no), parity (nulliparous or multiparous) and method of conception (spontaneous or in vitro fertilization) in the non-FGR pregnancies. Final regression models were then used to estimate the expected log_10_ levels of each biomarker (Supporting Information Table S2) and convert to a log_10_ multiple of their expected median level (MoM). The log_10_ MoM of PAPP-A was derived from the Down syndrome test which was corrected in our local population [22].

### Statistical analyses

Since there was no preliminary data on these biomarkers during pregnancy, the sample size was estimated based on effect size for the difference in mean level between FGR and non-FGR pregnancies. To detect a medium difference of 0.5 between two groups would require a minimum of 64 pregnancies for 80% power and Type I error of 5%.

Normality of the data was tested using the Kolmogorov-Smirnov test. Descriptive data were presented as mean ± SD or median (interquartile range [IQR]) for continuous variables, and as numbers and percentages for categorical variables. Univariate comparison between two groups was performed using student t test or Mann-Whitney U test, while comparison among three groups using analysis of variance (ANOVA) or Kruskal-Wallis test with Bonferroni post-hoc test for continuous variables, and chi-square tests or Fisher’s exact tests for categorical variables.

Pearson correlation coefficient (r) was measured between every two significantly differential biomarkers in the control group. Logistic regression was used to construct prediction models for FGR using the significantly differential biomarkers found on univariate analysis. The predictive performances of each single biomarker as well as combinations of biomarkers were assessed and compared with that of PAPP-A using area under receiver-operating characteristics curve (AUC). The Delong method was used to determine whether the difference between AUC was significant. A *P* value < 0.05 for the two-tailed test was considered statistically significant. Data analysis was performed by the statistical software package SPSS 28.0 (IBM Corp., Armonk, NY, USA).

## Results

### Clinical characteristics in different groups

From March 2019 to January 2022, we included 73 FGR cases, of which 26 were diagnosed as idiopathic early-onset FGR while 47 as idiopathic late-onset FGR. Maternal demographic characteristics and pregnancy outcomes of the FGR cases and their matched controls are compared in Table 3. There was no significant difference of maternal age, BMI, sampling gestational age, parity, method of conception, smoking status among the control, early-onset FGR and late-onset FGR group. The median gestational age of diagnosis of early-onset FGR was 28.6 (27.5–30.0) weeks, and was significantly earlier than late-onset FGR with 35.1 (34.0–36.0) weeks (*P* < 0.001). When compared to the control group, both the early-onset FGR and late-onset FGR groups had significantly lower median gestational ages at delivery (37.2 weeks, 37.7 weeks vs. 39.1 weeks, *P* < 0.001), lower median neonatal birthweight (2317 g, 2380 g vs. 3255 g, *P* < 0.001), and lower median BW z scores (-1.7, -1.7 vs. 0.0, *P* < 0.001). However, there was no difference between the early-onset FGR and the late-onset FGR groups. The rate of admission to neonatal intensive care units was significantly higher in both early-onset FGR and late-onset FGR groups compared to the control group (55.7%, 63.8% vs. 11.0%, *P* < 0.001). Other pregnancy outcomes, such as delivery mode, neonatal Apgar score, umbilical artery PH were similar among the groups.

**Table 3:** Maternal characteristics and pregnancy outcomes between the control group and the fetal growth restriction (FGR) groups

Variables		Controls (*n* = 73)	All FGR (*n* = 73)	*P* ^a^	Early-onset FGR (*n* = 26)	Late-onset FGR (*n* = 47)	*P* ^c^
Maternal age (years)		32.2 (29.6–34.2)	31.0 (28.0–34.0)	0.19	31.0 (28.8–34.2)	32.0 (28.0–34.0)	0.41
Maternal BMI (Kg/m^2^)		19.8 (19.0-22.2)	19.9 (18.8–22.1)	0.75	19.7 (18.9–21.8)	20.0 (18.8–22.2)	0.92
Nulliparous		43 (58.9)	42 (57.5)	0.87	12 (46.2)	30 (63.8)	0.35
Method of Conception							
Spontaneous		70 (95.9)	71 (97.3)	1.00^b^	25 (96.2)	46 (97.9)	1.00^b^
In vitro fertilization		3 (4.1)	2 (2.7)		1 (3.8)	1 (2.1)	
Smoking at conception		5 (6.8)	4 (5.5)	1.00^b^	1 (3.8)	3 (6.4)	1.00^b^
Gestation at sampling (weeks)		12.4 (12.1–12.6)	12.3 (11.9–12.6)	0.11	12.3 (12.0-12.6)	12.3 (11.9–12.6)	0.28
Gestation at diagnosis of FGR (weeks)		N.A.	34.0 (29.6–35.8)	N.A.	28.6 (27.5–30.0)	35.1 (34.0–36.0)	< 0.001
Gestation at delivery (weeks)		39.1 (38.3–39.9)^d, e^	37.4 (37.1–38.2)	< 0.001	37.2 (36.7–38.1)^d^	37.7 (37.1–38.3)^e^	< 0.001
Mode of Delivery							
Normal vaginal birth		46 (63.0)	49 (67.1)	0.82	20 (76.9)	29 (61.7)	0.70^b^
Assisted vaginal birth		9 (12.3)	7 (9.6)		1 (3.8)	6 (12.8)	
Cesarean delivery		18 (24.7)	17 (23.3)		5 (19.3)	12 (25.5)	
Neonatal sex							
Female		37 (50.7)	39 (53.4)	0.87	12 (46.2)	27 (57.4)	0.62
Male		36 (49.3)	34 (46.6)		14 (53.8)	20 (42.6)	
Birthweight (g)		3255 (3095–3358)^d, e^	2350 (2143–2505)	< 0.001	2317 (2080–2572)^d^	2380 (2195–2465)^e^	< 0.001
Birthweight Z score		0.0 (-0.3-0.4)^d, e^	-1.7 (-2.0-(-1.5))	< 0.001	-1.7 [-1.9-(-1.6)]^d^	-1.7 [-2.0-(-1.5)]^e^	< 0.001
Apgar Score							
<7 (1 min)		6 (8.2)	2 (2.7)	0.27	1 (3.8)	1 (2.1)	0.44^b^
≥7 (1 min)		67 (91.8)	71 (97.3)		25 (96.2)	46 (97.9)	
Umbilical Blood PH							
<7.20		14 (20.3)	9 (12.3)	0.20	1 (3.8)	8 (17.0)	0.15
≥7.20		55 (79.7)	64 (87.7)		25 (96.2)	39 (83.0)	
NICU Admission		8 (11.0)^d, e^	28 (38.4)	< 0.001	15 (55.7)^d^	30 (63.8)^e^	< 0.001

Data presented as median (interquartile range) for continuous variables and number (percentage) as categorical variables

*BMI*, body mass index; *FGR*, fetal growth restriction; *NICU*, neonatal intensive care unit; N.A, not applicable

^a^*P* value of Mann-Whitney U test or chi-square tests, ^b^Fisher’s exact test, ^c^Kruskal-Wallis test or chi-square tests; Bonferroni post-hoc test: ^d^early-onset FGR vs. control; ^e^late-onset FGR vs. control

### Angiogenic biomarkers’ levels in different groups

**Fig. 1:**
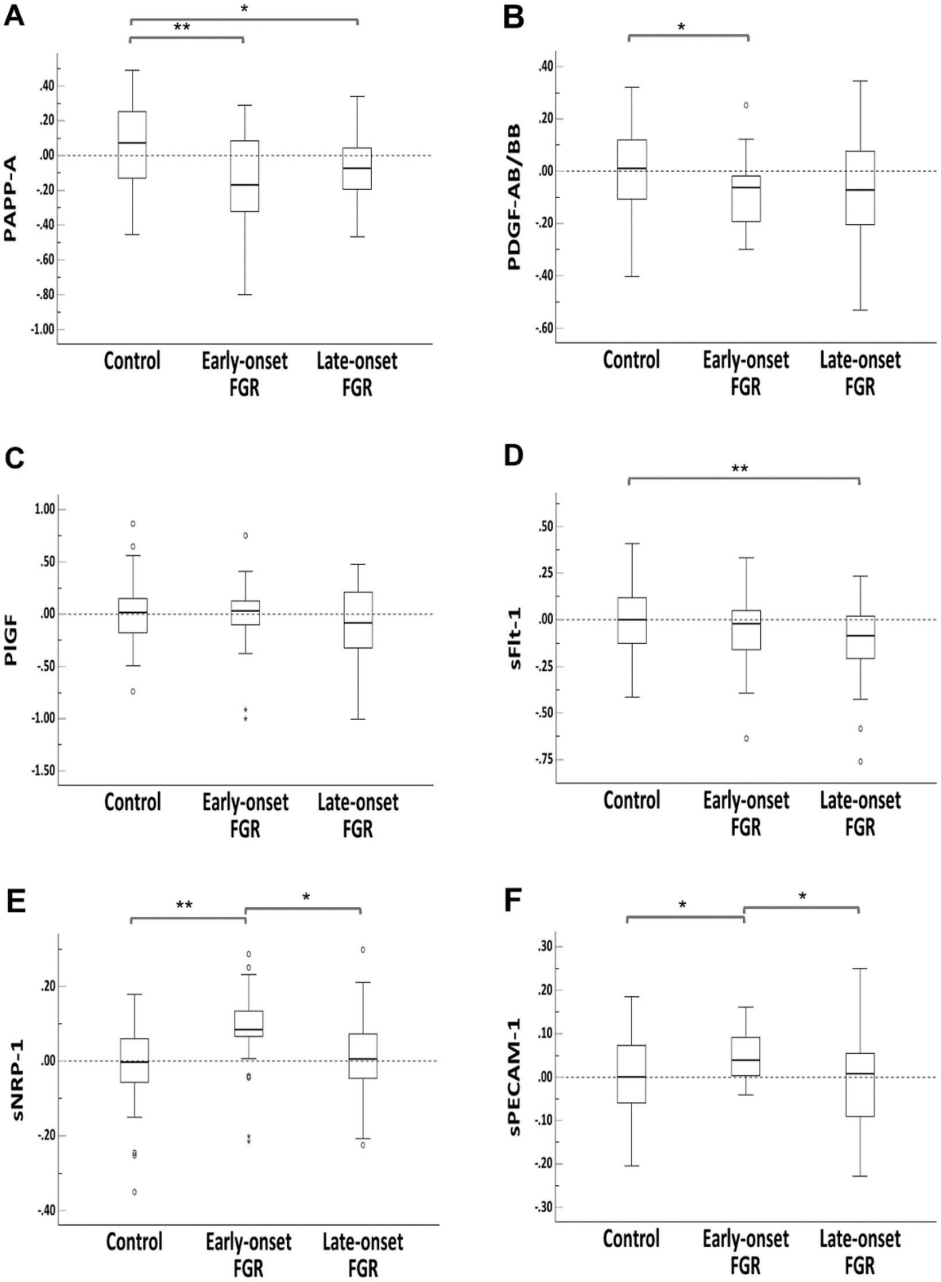
Comparison of Log_10_MoM of the angiogenic biomarkers between the control group and different fetal growth restriction groups. Box-whisker plots are presented for log_10_MoM of (**A**) PAPP-A, (**B**) PDGF-AB/BB, (**C**) PlGF, (**D**) sFlt-1, (**E**) sNRP-1, (**F**) sPECAM-1 in different groups. Single asterisk denotes *P* < 0.05, double asterisk denotes *P* < 0.001. Abbreviations: *FGR*, fetal growth restriction; *MoM*, multiple of median; *PAPP-A*, pregnancy-associated plasma protein A; *PDGF-AB/BB*, platelet-derived growth factor AB/BB; *PlGF*, placenta growth factor; *sFlt-1*, soluble fms-like tyrosine kinase-1; *sNRP-1*, soluble neuropilin-1; *sPECAM-1*, soluble platelet and endothelial cell adhesion molecule 1

Among the 36 angiogenic biomarkers, two biomarkers’ concentrations, Vascular Endothelial Growth Factor A (VEGF-A) and Fibroblast Growth Factor 1 (FGF-1), were lower while Angiopoietin 2 (ANG-2) concentration was higher than the panels’ detection range, and hence they were excluded from our study. After adjusting for maternal confounding factors (gestational age, maternal age, height, weight, smoking, parity and method of conception), we identified several new biomarkers, including soluble neuropilin-1 (sNRP-1), soluble platelet and endothelial cell adhesion molecule 1 (sPECAM-1), and platelet-derived growth factor AB/BB, log_10_MoM of which were different between the overall FGR group and the control group (Table 4). On subgroup analysis, log_10_MoM of sNRP-1 (0.08 ± 0.11 vs. 0.00 ± 0.09, adjusted *P* < 0.001) and log_10_MoM of sPECAM-1 (0.05 ± 0.06 vs. 0.00 ± 0.09, adjusted *P* < 0.05) were significantly higher while log_10_MoM of PDGF-AB/BB was significantly lower (-0.08 ± 0.13 vs. 0.00 ± 0.16, adjusted *P* < 0.05) in the early-onset FGR group. However, they were not different between the late-onset FGR group and the control group. Log_10_MoM of PAPP-A was significantly lower in both early-onset (-0.15 ± 0.28 vs. 0.05 ± 0.23, adjusted *P* < 0.001) and late-onset FGR (-0.07 ± 0.18 vs. 0.05 ± 0.23, adjusted *P* < 0.05) when compared to control group. Log_10_MoM of sFlt-1 (-0.12 ± 0.21 vs. 0.00 ± 0.17, adjusted *P* < 0.001) was lower in the late-onset FGR group when compared to control group, but it was not different between early-onset FGR group and the control group. Log_10_MoM of PlGF was lower in FGR group, but the difference was not statistically significant (Table 4; Fig. 1). The other 28 biomarkers had no significant differences between the groups (Supporting Information Table S3).

**Table 4:** Comparison of first trimester maternal serum levels of different biomarkers between the control group and the fetal growth restriction (FGR) groups

Log_10_MoM of biomarkers’	Control	All FGR	*P* ^a^	Early-onset FGR	Late-onset FGR	*P* ^b^
PAPP-A	0.05 ± 0.23^c, d^	-0.10 ± 0.22	< 0.001	-0.15 ± 0.28^c^	-0.07 ± 0.18^d^	< 0.001
PDGF-AB/BB	0.00 ± 0.16^c^	-0.07 ± 0.17	0.009	-0.08 ± 0.13^c^	-0.07 ± 0.19	0.032
PlGF	0.00 ± 0.27	-0.08 ± 0.37	0.11	-0.02 ± 0.36	-0.12 ± 0.38	0.13
sFlt-1	0.00 ± 0.17^d^	-0.10 ± 0.21	0.003	-0.06 ± 0.21	-0.12 ± 0.21^d^	0.007
sNRP-1	0.00 ± 0.09^c^	0.04 ± 0.11	0.042	0.08 ± 0.11^c, e^	0.01 ± 0.11^e^	0.004
sPECAM-1	0.00 ± 0.09^c^	0.01 ± 0.09	0.31	0.05 ± 0.06^c, e^	-0.01 ± 0.10^e^	0.023

Data presented as mean ± SD

*FGR*, fetal growth restriction; *MoM*, multiple of median; *PAPP-A*, pregnancy-associated plasma protein A; *PDGF-AB/BB*, platelet-derived growth factor AB/BB; *PlGF*, placenta growth factor; *sFlt-1*, soluble fms-like tyrosine kinase-1; *sNRP-1*, soluble neuropilin-1; *sPECAM-1*, soluble platelet and endothelial cell adhesion molecule 1

^a^*P* value of *student t test*, ^*b*^*P* value of analysis of variance (ANOVA) test; ^c^ early onset FGR vs. control; ^d^ late-onset FGR vs. control; ^e^ early-onset vs. late-onset FGR

### Prediction performance of angiogenic biomarkers for FGR

**Fig. 2:**
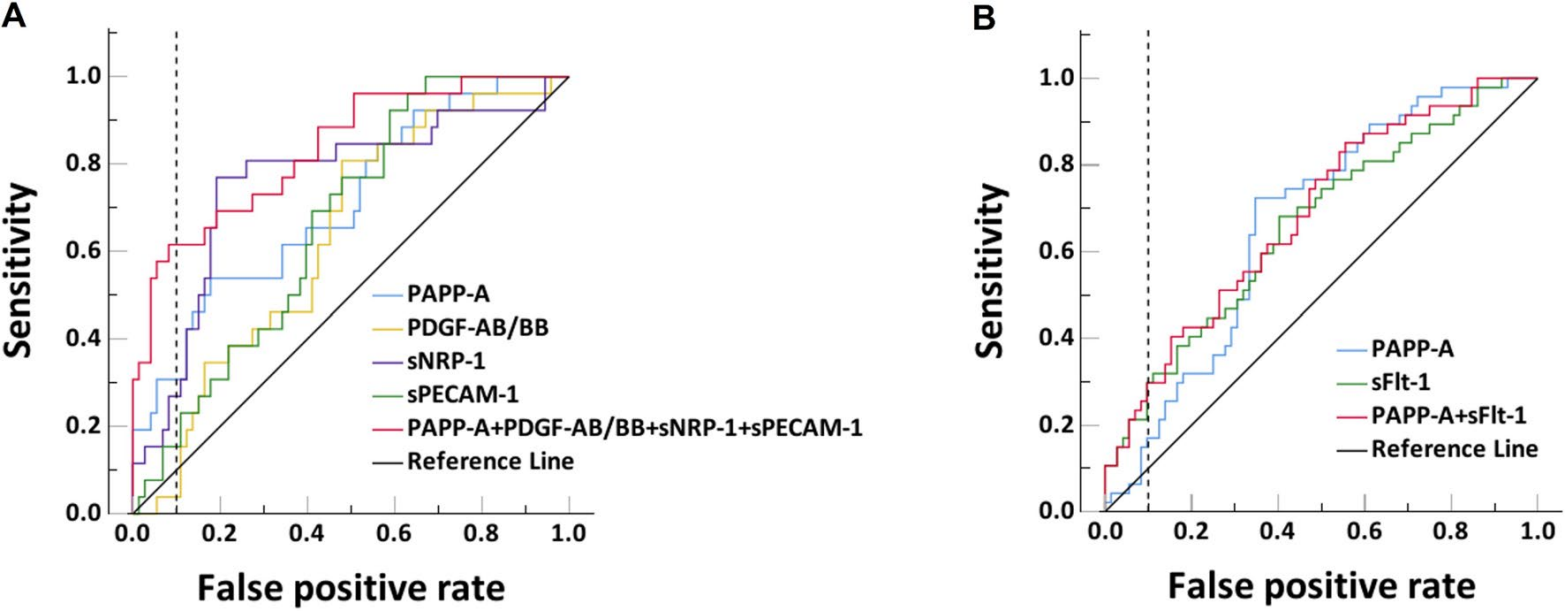
The receiver operating characteristics curves of angiogenic biomarkers for prediction of early-onset (**A**) and late-onset (**B**) fetal growth restriction at 11–13 weeks. Dash vertical lines indicate 10% false positive rate. Abbreviations: *FGR*, fetal growth restriction; *PAPP-A*, pregnancy-associated plasma protein A; *PDGF-AB/BB*, platelet-derived growth factor AB/BB; *sFlt-1*, soluble fms-like tyrosine kinase-1; *sNRP-1*, soluble neuropilin-1; *sPECAM-1*, soluble platelet and endothelial cell adhesion molecule 1

The Pearson correlation coefficients between every two significantly differential biomarkers are shown in Supporting Information Table S4. Only PAPP-A and sFlt-1 (*r* = 0.55, *P* < 0.001), PDGF-AB/BB and sPECAM-1 (*r* = 0.27, *P* = 0.02) had moderate or weak correlations. No obvious collinearity would be a concern when we combine the biomarkers for prediction as multicollinearity only affects the highly correlated variables. The predictive performances of the individual markers and their combinations for early-onset FGR are shown in Table 5; Fig. 2A. The AUC (95%CI) for early-onset FGR was the highest in sNRP-1: 0.76 (0.64–0.87), followed by PAPP-A: 0.71(0.59–0.83), then sPECAM-1: 0.67 (0.56–0.77) and PDGF-AB/BB: 0.63 (0.52–0.75), but their differences from PAPP-A had not reached a statistical significance. The AUCs of combinations of any two biomarkers and any three biomarkers ranged from 0.72 to 0.82 but were not statistically higher than that of PAPP-A. The combination of the three angiogenic markers with PAPP-A achieved the highest AUC of 0.83 (0.74–0.93) which was statistically higher than that of PAPP-A (*P* < 0.001). Its sensitivity of 61.5% was double of PAPP-A (30.8%) at a 10% false positive rate with significant difference (*P* < 0.05) (Table 5; Fig. 2A). For late-onset FGR, AUCs (95%CI) of sFlt-1 and PAPP-A were similar, which were 0.66 (0.56–0.76) and 0.66 (0.57–0.76), respectively. The combination of sFlt-1 and PAPP-A achieved an AUC of 0.68 (0.59–0.78), but the sensitivity was 29.8% at 10% false positive rate (Table 6; Fig. 2B).

**Table 5:** The prediction performance of angiogenic markers for early-onset fetal growth restriction

Log_10_MoM of biomarkers	AUC (95%CI)	*P* ^a^	Sensitivity (%) (95% CI) at 10% FPR
PAPP-A	0.71 (0.59–0.83)^b^	< 0.001	30.8 (11.8–49.8)^c^
PDGF-AB/BB	0.63 (0.52–0.75)	0.046	3.8 (0.0-11.8)
sNRP-1	0.76 (0.64–0.87)	< 0.001	26.9 (8.7–45.2)
sPECAM-1	0.67 (0.56–0.77)	0.013	15.4 (1.0-30.2)
PAPP-A + PDGF-AB/BB	0.72 (0.60–0.83)	< 0.001	34.6 (15.0-54.2)
PAPP-A + sNRP-1	0.78 (0.67–0.89)	< 0.001	50.0 (29.4–70.6)
PAPP-A + sPECAM-1	0.76 (0.66–0.86)	< 0.001	30.8 (11.8–49.8)
PDGF-AB/BB + sNRP-1	0.80 (0.69–0.91)	< 0.001	61.5 (41.5–81.6)
PDGF-AB/BB + sPECAM-1	0.76 (0.66–0.86)	< 0.001	15.4 (1.0-30.2)
sNRP-1 + sPECAM-1	0.76 (0.66–0.87)	< 0.001	38.5 (18.4–58.5)
PAPP-A + PDGF-AB/BB + sNRP-1	0.79 (0.68–0.90)	< 0.001	57.7 (37.3–78.0)
PAPP-A + PDGF-AB/BB + sPECAM-1	0.78 (0.69–0.88)	< 0.001	38.5 (18.4–58.5)
PAPP-A + sNRP-1 + sPECAM-1	0.80 (0.70–0.90)	< 0.001	46.2 (25.6–66.7)
PDGF-AB/BB + sNRP-1 + sPECAM-1	0.82 (0.73–0.92)	< 0.001	57.7 (37.3–78.0)
PAPP-A + PDGF-AB/BB + sNRP-1 + sPECAM-1	0.83 (0.74–0.93)^b^	< 0.001	61.5 (41.5–81.6)^c^

*AUC*, area under receiver operating characteristics curve; *FPR*, false positive rate; *MoM*, multiple of median; *PAPP-A*, pregnancy-associated plasma protein A; *PDGF-AB/BB*, platelet-derived growth factor; AB/BB; *sNRP-1*, soluble neuropilin-1; *sPECAM-1*, soluble platelet and endothelial cell adhesion molecule 1

^a^*P* value of AUC; ^b^paired AUC DeLong test *P* < 0.05; ^c^Fisher’s exact test *P* < 0.05

**Table 6:** The prediction performance of angiogenic markers for late-onset fetal growth restriction

Log_10_MoM of biomarkers	AUC (95%CI)	*P* ^a^	Sensitivity (%) (95% CI) at 10%FPR
PAPP-A	0.66 (0.57–0.76)	0.001	17.0 (5.9–28.2)
sFlt-1	0.66 (0.56–0.76)	0.003	29.8 (16.2–43.4)
PAPP-A + sFlt-1	0.68 (0.59–0.78)	< 0.001	29.8 (16.2–43.4)

*AUC*, area under receiver operating characteristics curve; *FPR*, false positive rate; *MoM*, multiple of median; *PAPP-A*, pregnancy-associated plasma protein A; *sFlt-1*, soluble fms-like tyrosine kinase-1

^a^*P* value of AUC

## Discussion

In this first study on comprehensive profiling of first trimester maternal serum angiogenic biomarkers for FGR, we demonstrated the association between early-onset FGR and three new biomarkers, in addition to PAPP-A. During 11 to 13 weeks, the maternal serum levels of two anti-angiogenic biomarkers, sNRP-1 and sPECAM-1, were higher, while that of the pro-angiogenic biomarker PDGF-AB/BB was lower in those pregnancies subsequently developed early-onset FGR. For late-onset FGR, only PAPP-A and sFlt-1 were found to be lower in the late-onset subgroup. This differential finding may indicate a different patho-mechanism between early-onset and late-onset FGR.

Among the three new biomarkers, we found that a high sNRP-1 level had the highest AUC. sNRP-1 is the cleaved and soluble form of the extracellular part of NRP-1, a transmembrane receptor that plays a crucial role in sprouting angiogenesis as a tip cell function [23]. NRP-1 acts as a co-receptor for VEGFA, dominantly VEGF_165_, to enhance VEGFR2 activation and signaling to promote endothelial cell proliferation and migration. On the other hand, sNRP-1 acts as an antagonist of NRP1, and functions as anti-angiogenic protein, inhibiting VEGF binding to endothelial cell and signal transduction. In previous placental studies, NRP-1 expression was found in in human decidua and trophoblast in all three trimesters, indicating its crucial role in embryonic implantation and placentation process [24]. NRP-1 was shown to be down-regulated at RNA and protein level in FGR placentae [25]. However, a recent study found that sNRP-1 was decreased in maternal plasma in FGR complicated pathologic umbilical artery Doppler, which was contrary to the previous placental studies and our study [26]. One of the possible reasons for the conflicting results may be that this study measured sNRP-1 concentration at 24–40 gestation weeks [26], while in our study it was measured at 11–13 weeks, during when branching angiogenesis is predominant, and angiogenesis becomes non-branching afterwards. Secondly, we differentiated early-onset and late-onset FGR in our analysis. Our finding of an increased sNRP-1 level at 11–13 weeks may indicate a compromised branching angiogenesis of fetoplacental vascular development in the pathophysiology of early-onset FGR [27].

sPECAM-1 is also a soluble form and antagonist of PECAM-1, which facilitates the process of angiogenesis, including endothelial cell signal transduction, migration, proliferation and cell-cell junction formation [28]. PECAM-1 were expressed on the endothelium of villi and decidua vessels in placenta [29, 30]. Previous placental studies demonstrated that PECAM-1 showed no change in placentae in FGR [30], and either unchanged [29, 30] or reduced [31] in pre-eclamptic placentae. A recent study also showed no significant change in the serum level of PECAM-1 in women with pre-eclampsia [32]. Our study is the first to investigate maternal circulating PECAM-1 level in FGR, and found that it was higher in the first trimester, revealing an impairment of angiogenesis contributing to the etiology of FGR.

The PDGF family consists of five different proteins, PDGF-AA, PDGF-AB, PDGF-BB, PDGF-CC, PDGF-DD. PDGFs, acting as a pro-angiogenic factor, could stimulate cell proliferation, migration and angiogenesis, playing a crucial role in different physiological and pathological process, including embryonic development, blood vessel formation, wound healing and cancer [33]. Maternal levels of PDGFs was studied in pre-eclamptic patients with conflicting results. While some studies showed an increased level [34, 35], others did not [36]. No study had investigated the role of PDGF in FGR. Our finding of a lower PDGF-AB/BB level among mothers with early-onset FGR suggests that placental angiogenesis may have been dysfunctional since the first trimester.

PAPP-A is a well-known protein in promoting fetal growth and a low first trimester PAPP-A level is associated with FGR [12, 13, 37, 38]. Our study further revealed that a low PAPP-A level was associated with both early-onset and late-onset FGR and was distinct from the three angiogenic biomarkers which were only associated with early-onset FGR.

sFlt-1 is the soluble form of VEGFR-1 generated from alternative splicing of the FLT1 gene. It acts as a decoy receptor of PlGF and VEGF, inhibiting and signaling their own receptors on endothelial cells of normal angiogenesis and vascular development. Studies on the first trimester level of sFlt-1 among FGR pregnancies were not consistent. While some studies showed a higher sFlt-1 level [10], others found no difference [39, 40] or a lower level [41, 42]. A systematic review of eight studies comparing 762 small for gestational age cases and 1316 controls found that there was no significant difference in the first trimester level of sFlt-1 between the two groups [11]. Our finding is distinct as we differentiated early-onset and late-onset FGR fetuses, and sFlt-1 was significantly decreased only in late-onset FGR group. Our result suggests that the patho-mechanism of the two phenotypes may be different.

PlGF is a pro-angiogenic protein expressed in placentae and is detectable in maternal circulation. A low first trimester maternal circulating PlGF level is known to be associated with subsequent development of pre-eclampsia and FGR. Yet its predictive value is more supreme in pre-eclampsia than isolated FGR [38, 43]. In a recent systematic review which included eight studies of 1055 small for gestational age cases and 3134 controls, it was found that the difference in PlGF level between small for gestational age group and control group before 14 gestational weeks was minimal at -5.2 (-6.6 to -3.7) pg/ml, and 3 of these 8 studies failed to find a significant difference between two groups [11, 39–41]. In our study, we also found a lower PlGF level in the FGR group, but the difference had not reached a statistical significance. This could be due to the exclusion of subjects with hypertensive disorders in pregnancy or at high risk of pre-eclampsia, in which PlGF would be much lower. Our results may also indicate a different patho-mechanism between FGR and pre-eclampsia.

Early-onset FGR is associated with significant morbidity and mortality, yet the prediction by the existing clinical, sonographic or biochemical methods is not good enough for routine practice. In particular, the reported sensitivity of PAPP-A for overall FGR ranged from 6.9 to 27.8% at a false positive rate of 10% [10, 12, 38]. However, we found that when it was combined with the three new angiogenic biomarkers, the sensitivity of early-onset FGR in first trimester can be very much improved from 30.8 to 61.5%, at a false positive rate of 10%. The screened positive (high risk) cases may then be triaged for follow-up sonographic monitoring. This strategy may be more cost-effective than universal sonographic screening, and may allow early diagnosis and management of FGR. The multiplexed spheres-based assay we used in our study can measure multiple angiogenic biomarkers simultaneously at a cost of about USD120. Further large-scale prospective studies are required to confirm the accuracy and cost-effectiveness of this new strategy.

Our study has several strengths. Firstly, the definition of FGR was based on the prenatal Delphi criteria, and all the cases were further confirmed after delivery with actual birthweight. We also followed Delphi criteria to use 32 gestational weeks to stratified FGR into early-onset and late-onset, and revealed the differential results. In addition, we excluded cases with known maternal and fetal causes. In particular, those cases complicated with hypertensive disorders in pregnancy and those screened as high risk for pre-eclampsia, aiming at a distinguished group of FGR without apparent causes. We also excluded cases with aspirin prophylaxis to avoid bias by any drug effect. By doing so, we revealed that PlGF may not be a significant marker for non-pre-eclamptic related FGR. This may indicate that different pathological mechanisms are involved, and also explain why a recent systematic review did not find a significant difference in PlGF level between small for gestational age group and control group before 14 gestational weeks [11]. We also excluded intrauterine deaths and miscarriages, which were potentially the worst cases. Yet we were still able to demonstrate significant differences between FGR groups and the control group. Finally, this is an exploratory case-control study so the sample size was small. In future, a larger prospective cohort study is required to externally verify our findings, and to develop a prediction algorithm combining maternal factors, uterine artery Doppler and other risk factors.

## Conclusions

We found three new first trimester angiogenic biochemical predictors for subsequent development of early-onset FGR. During 11–13 weeks, the maternal serum levels of sNRP-1 and sPECAM-1 were increased, while PDGF-AB/BB was decreased in pregnancies which later developed early-onset FGR. The sensitivity for early-onset FGR may be improved from 30.8% when using PAPP-A alone, to 61.5% when combining PAPP-A with the three new markers. For late-onset FGR, sFlt-1 and PAPP-A levels were lower, but their predictive values were poor.

## Electronic supplementary material

Below is the link to the electronic supplementary material.

Supplementary Material 1

## Data Availability

The datasets used and/or analysed during the current study are available from the corresponding author on reasonable request.

## Abbreviations

AUC: Area under receiver-operating characteristics curve
BW: Birthweight
FGR: Fetal growth restriction
PDGF: Platelet-derived growth factor
PlGF: Placental growth factor
PAPP-A: Pregnancy-associated plasma protein-A
sFlt-1: Soluble fms-like tyrosine kinase 1
sNRP-1: Soluble neuropilin-1
sPECAM-1: Soluble platelet and endothelial cell adhesion molecule 1
