# Supplementary material for: New first trimester circulating angiogenic biomarkers in predicting early-onset and late-onset fetal growth restriction: a case-control study
Source: BMC Pregnancy Childbirth. 2025 May 10;25:562. doi: 10.1186/s12884-025-07558-4 (PMC12066072; doi:10.1186/s12884-025-07558-4)
Supplement: Supplementary file 1 — Supplementary Material 1 [file 12884_2025_7558_MOESM1_ESM.docx]

**TABLE S1 The limit of detection, intra-assay and inter-assay coefficients of variation for each angiogenesis biomarker**

| Biomarkers | Limit of detection (minimum detectable concentration, pg/ml) | intra-assay coefficients of variation (%) | inter-assay coefficients of variation (%) |
| --- | --- | --- | --- |
| ANG-2 | 3.0 | <10 | <20 |
| ANGST | 348.3 | <10 | <15 |
| BMP-9 | 1.2 | <10 | <20 |
| EGF | 1.0 | <10 | <20 |
| ENG | 17.0 | <10 | <20 |
| ET-1 | 1.4 | <10 | <20 |
| FGF-1 | 4.6 | <10 | <20 |
| FGF-2 | 8.6 | <10 | <20 |
| FS | 11.1 | <10 | <20 |
| G-CSF | 5.4 | <10 | <20 |
| HB-EGF | 0.4 | <10 | <20 |
| HGF | 8.5 | <10 | <20 |
| IL-8 | 0.2 | <10 | <20 |
| LEP | 42.8 | <10 | <20 |
| OPN | 29.6 | <10 | <15 |
| PDGF-AB/BB | 6.4 | <10 | <15 |
| PlGF | 0.7 | <10 | <20 |
| sAXL | 2.7 | <10 | <15 |
| sc-Met | 10.4 | <10 | <15 |
| sc-Kit | 10.5 | <10 | <15 |
| sEGFR | 16.0 | <10 | <15 |
| sFlt-1 | 2.1 | <10 | <15 |
| sFlt-4 | 82.9 | <10 | <15 |
| sHER2 | 5.0 | <10 | <15 |
| sHER3 | 6.1 | <10 | <15 |
| sIL-6Ra | 7.4 | <10 | <15 |
| sNRP-1 | 54.9 | <10 | <15 |
| sPECAM-1 | 6.3 | <10 | <15 |
| sSELE | 116.6 | <10 | <15 |
| sTie-2 | 12.2 | <10 | <15 |
| su-PAR | 36.9 | <10 | <15 |
| sVEGFR2 | 19.2 | <10 | <15 |
| TSP-2 | 23.5 | <10 | <15 |
| VEGF-A | 8.1 | <10 | <20 |
| VEGF-C | 7.6 | <10 | <20 |
| VEGF-D | 1.9 | <10 | <20 |

**TABLE S2 Equations of expected log_10_ values of each angiogenesis biomarker**

| Expected log10 biomarkers’ value | Equation | R^2^ | *P* |
| --- | --- | --- | --- |
| ANGST | 4.386534 +0.010574 * age (years) | 0.112 | 0.004 |
| BMP-9 | 2.056037 + 0.014502 * age (years) | 0.060 | 0.036 |
| EGF | Constance = 2.1265 | N.A. | N.A. |
| ENG | 4.007195 - 0.010123 * weight (kg) | 0.145 | <0.001 |
| ET-1 | Constance = 1.1770 | N.A. | N.A. |
| FGF-2 | Constance = 2.1526 | N.A. | N.A. |
| FS | 0.863191 + 0.181104 * GA (weeks) | 0.097 | 0.008 |
| G-CSF | -0.493441 + 0.179749 * GA (weeks)+0.015242* age (years) | 0.158 | 0.003 |
| HB-EGF | 1.826522 – 0.267418 * 1 (if smoking) | 0.084 | 0.013 |
| HGF | 4.755909 - 0.013037 * height (cm) | 0.067 | 0.028 |
| IL-8 | Constance = 0.4579 | N.A. | N.A. |
| LEP | 6.206008 +0.028487 * weight (kg)-0.022177 *height (cm) | 0.411 | <0.001 |
| OPN | 3.147967-0.209375 * 1 (if multiparous) | 0.150 | <0.001 |
| PDGF-AB/BB | 3.540797 + 0.007957* weight (kg) | 0.070 | 0.027 |
| PlGF | -2.337637+ 0.305997 *GA (weeks) | 0.206 | <0.001 |
| sAXL | Constance =3.1812 | N.A. | N.A. |
| sc-Met | Constance = 5.0458 | N.A. | N.A. |
| sc-Kit | Constance = 4.5195 | N.A. | N.A. |
| sEGFR | Constance = 3.8355 | N.A. | N.A. |
| sFlt-1 | 3.986685 - 0.007565 * weight (kg) | 0.073 | 0.023 |
| sFlt-4 | Constance =3.2012 | N.A. | N.A. |
| sHER2 | Constance =3.8934 | N.A. | N.A. |
| sHER3 | Constance =3.8984 | N.A. | N.A. |
| sIL-6Ra | Constance = 4.7992 | N.A. | N.A. |
| sNRP-1 | Constance = 4.9519 | N.A. | N.A. |
| sPECAM-1 | Constance =3.5551 | N.A. | N.A. |
| sSELE | Constance = 4.8613 | N.A. | N.A. |
| sTie-2 | 3.794890 – 0.275857 * 1 (if smoking) | 0.079 | 0.017 |
| su-PAR | Constance = 3.6256 | N.A. | N.A. |
| sVEGFR2 | Constance = 4.2191 | N.A. | N.A. |
| TSP-2 | 4.639728 – 0.012905 * age (years) - 0.007722 * weight (kg) | 0.144 | 0.006 |
| VEGF-C | 4.306086 - 0.007403 * height (cm)+0.009126*age (years) | 0.156 | 0.004 |
| VEGF-D | Constance = 2.9656 | N.A. | N.A. |

**TABLE S3 Comparisons of insignificantly differential angiogenesis biomarkers between the control group and different fetal growth restriction groups**

| Log_10_ MoM of biomarkers | Control | Total FGR | *P^a^* | Early-onset FGR | Late-onset FGR | *P^b^* |
| --- | --- | --- | --- | --- | --- | --- |
| ANGST | 0.00 ± 0.12 | -0.01 ± 0.17 | 0.62 | -0.01 ± 0.17 | -0.01 ± 0.17 | 0.86 |
| BMP-9 | 0.00 ± 0.23 | 0.05 ± 0.24 | 0.19 | 0.06 ± 0.22 | 0.05 ± 0.26 | 0.42 |
| EGF | -0.10 ± 0.31 | -0.10 ± 0.31 | 0.98 | -0.02 ± 0.26 | -0.14 ± 0.33 | 0.35 |
| ENG | 0.00 ± 0.16 | -0.05 ± 0.22 | 0.15 | -0.02 ± 0.23 | -0.06 ± 0.22 | 0.25 |
| ET-1 | 0.00 ± 0.21 | 0.01 ± 0.23 | 0.68 | 0.06 ± 0.24 | -0.02 ± 0.22 | 0.50 |
| FGF-2 | 0.00 ± 0.19 | -0.07 ± 0.27 | 0.09 | -0.08 ± 0.24 | -0.06 ± 0.32 | 0.23 |
| FS | 0.00 ± 0.23 | -0.04 ± 0.26 | 0.33 | -0.09 ± 0.32 | -0.01 ± 0.22 | 0.28 |
| G-CSF | 0.00 ± 0.23 | 0.04 ± 0.26 | 0.44 | 0.05 ± 0.31 | 0.02 ± 0.24 | 0.70 |
| HB-EGF | 0.00 ± 0.22 | -0.06 ± 0.31 | 0.17 | -0.06 ± 0.31 | -0.06 ± 0.30 | 0.39 |
| HGF | 0.00 ± 0.25 | 0.03 ± 0.29 | 0.52 | 0.05± 0.28 | 0.02 ± 0.29 | 0.70 |
| IL-8 | -0.01 ± 0.22 | 0.05 ± 0.35 | 0.33 | 0.15 ± 0.37 | -0.02 ± 0.32 | 0.13 |
| LEP | 0.00 ± 0.19 | 0.01 ± 0.26 | 0.77 | 0.04± 0.32 | -0.01 ± 0.22 | 0.62 |
| OPN | 0.00 ± 0.25 | 0.05 ± 0.29 | 0.30 | 0.02 ± 0.30 | 0.06 ± 0.28 | 0.47 |
| sAXL | -0.01± 0.14 | -0.04 ± 0.21 | 0.34 | -0.03 ± 0.23 | -0.05 ± 0.20 | 0.60 |
| sc-Met | -0.04 ± 0.15 | -0.03 ± 0.17 | 0.77 | -0.02 ± 0.15 | -0.04 ± 0.18 | 0.88 |
| sc-Kit | 0.00 ± 0.09 | -0.02 ± 0.15 | 0.24 | 0.00 ± 0.11 | -0.04 ± 0.16 | 0.21 |
| sEGFR | -0.02 ± 0.23 | -0.04 ± 0.22 | 0.64 | -0.10 ± 0.22 | 0.00 ± 0.22 | 0.19 |
| sFlt-4 | 0.01 ± 0.31 | 0.00± 0.37 | 0.86 | -0.04 ± 0.44 | 0.03 ± 0.32 | 0.68 |
| sHER2 | -0.01 ± 0.08 | 0.00 ± 0.09 | 0.84 | 0.03 ± 0.07 | -0.02 ± 0.09 | 0.07 |
| sHER3 | -0.06 ± 0.25 | -0.05 ± 0.25 | 0.81 | -0.02 ± 0.19 | -0.07 ± 0.28 | 0.71 |
| sIL-6Ra | 0.01 ± 0.13 | 0.01 ± 0.11 | 0.88 | 0.01 ± 0.11 | 0.00 ± 0.12 | 0.92 |
| sSELE | -0.03 ± 0.19 | -0.04 ± 0.17 | 0.95 | 0.00 ± 0.15 | -0.06 ± 0.18 | 0.35 |
| sTie-2 | 0.00 ± 0.24 | 0.03 ± 0.28 | 0.51 | 0.08 ± 0.29 | 0.00 ± 0.27 | 0.40 |
| su-PAR | 0.01 ± 0.19 | 0.02 ± 0.19 | 0.86 | 0.03 ± 0.19 | 0.01 ± 0.19 | 0.90 |
| sVEGFR2 | -0.01 ± 0.10 | 0.00 ± 0.11 | 0.92 | -0.01 ± 0.11 | 0.00 ± 0.11 | 0.93 |
| TSP-2 | 0.00 ± 0.18 | 0.03 ± 0.23 | 0.36 | 0.02 ± 0.21 | 0.04 ± 0.24 | 0.64 |
| VEGF-C | 0.00 ± 0.13 | 0.01 ± 0.19 | 0.68 | 0.04 ± 0.17 | -0.01 ± 0.20 | 0.40 |
| VEGF-D | 0.00 ± 0.27 | -0.06 ± 0.36 | 0.27 | -0.13 ± 0.36 | -0.02 ± 0.35 | 0.22 |

^a^*P* value of *student t test, ^b^P* value of analysis of variance (ANOVA) test;

**TABLE S4 Pearson correlation coefficients between biomarkers in control group**

| **Log_10_MoM of biomarkers’** |  |  |  |  |  |
| --- | --- | --- | --- | --- | --- |
| **PAPP-A** | 1 |  |  |  |  |
| **PDGF-AB/BB** | 0.067 | 1 |  |  |  |
| **sFlt-1** | 0.55^a^ | 0.23 | 1 |  |  |
| **sNRP-1** | 0.16 | 0.15 | 0.20 | 1 |  |
| **sPECAM-1** | -0.063 | 0.27^a^ | 0.17 | 0.21 | 1 |
|  | **PAPP-A** | **PDGF-AB/BB** | **sFlt-1** | **sNRP-1** | **sPECAM-1** |

*PAPP-A*, pregnancy-associated plasma protein A; *PDGF-AB/BB*, platelet-derived growth factor AB/BB; *sFlt-1*, soluble fms-like tyrosine kinase-1; *sNRP-1*, soluble neuropilin-1; *sPECAM-1*, soluble platelet and endothelial cell adhesion molecule 1

^a^*P* <0.05
